# Moderate dose of watercress and red radish does not reduce oxygen consumption during graded exhaustive exercise

**Published:** 2014

**Authors:** Abbas Meamarbashi, Meysam Alipour

**Affiliations:** 1 *Department of Physical Education and Sports Sciences, University of Mohaghegh Ardabili, Ardabil, I. R. Iran*

**Keywords:** Exercise, Nitrate, Red radish, Watercress

## Abstract

**Objective: **Very recent studies have reported positive effects of dietary nitrate on the oxygen consumption during exercise. This research aimed to study the effect of moderate dose of high-nitrate vegetables, watercress (*Nasturtium officinale*) and red radish (*Raphanus sativus*) compared with a control group on the incremental treadmill exercise test following a standard Bruce protocol controlled by computer.

**Materials and Methods:** Group 1 consumed 100 g watercress (n=11, 109.5 mg nitrate/day), and group 2 consumed 100 g red radish (n=11, mg 173.2 mg nitrate/day) for seven days, and control group (n=14) was prohibited from high nitrate intake.

**Results:** During exercise, watercress group showed significant changes in the maximum values of Respiratory Exchange Ratio (RER) (p<0.05), End-Tidal O_2_ Fraction (FETO_2_) (p<0.05), and energy consumption from carbohydrate (p<0.01). Red radish group had a significant increase in the VCO_2_ (p<0.01), RER (p<0.01), VT (p<0.05), VCO_2_/kg (p<0.05), and energy consumption from carbohydrates (p<0.01). When all groups in the same workload were normalized by the subject’s body mass, watercress had a significant increase in the total expired CO_2 _(p<0.05), RER (p<0.05), FETO_2_ (p<0.05), and energy consumption from carbohydrates (p<0.05) compared with the control group. Similar comparison between red radish and control group revealed a significant increase during pre-test in the total CO_2_ production (p<0.05), VCO_2_ (p<0.05), RER (p<0.01), VT (p<0.05), and VCO_2_/kg (p<0.05).

**Conclusion** **: **Current results indicate higher carbon dioxide production in the experimental groups in the same workload. This might have a negative impact on the exercise performance. Further investigations with controlled exercise program will be necessary.

## Introduction

Green leafy vegetables are rich sources of nitrate. However, during the last decade its importance in biological processes has been increasingly appreciated (Gladwin, et al., 2005 ▶, Lundberg, et al., 2008 ▶, van Faassen, et al., 2009 ▶). Several lines of evidence indicate that the nitrate-nitrite-NO pathway is involved in regulation of blood flow, blood pressure (Larsen, et al., 2006 ▶), cell signaling, glucose homeostasis (Carlstrom, et al., 2010 ▶), and tissue response to hypoxia (Gladwin, et al., 2005 ▶, Lundberg, et al., 2008 ▶, van Faassen, et al., 2009 ▶). 

It should be noted that nitrate has potential carcinogenic effects and is responsible for blue baby syndrome, cancer and occasional intoxications (EFSA, 2008 ▶). Recent scientific evidence has provided clues to the molecular interaction between nitrite and heme proteins in the blood and tissues, as well as the role of nitrite in hypoxic vasodilatation, and protective effects of nitrite against ischemia/reperfusion injury. From the biochemical point of view on the nitrite and nitric oxide (NO), it is persuading to assume that nitrite acts as a source of NO when functioning as a vasodilator (Butler and Feelisch, 2008 ▶). Nitrate was speculated to modulate the mitochondrial function (Shiva, et al., 2007 ▶), lowering blood pressure (Larsen, et al., 2006 ▶), enhance gastric mucosal defense mechanisms (Petersson, et al., 2007 ▶), and reduces the oxygen cost of exercise (Lansley, et al., 2011 ▶, Larsen, et al., 2011 ▶, Larsen, et al., 2010 ▶). Recent findings about the possibility of enhancing performance by using nitrate attracted a great interest among the sports physiologists. 

**Figure 1:**
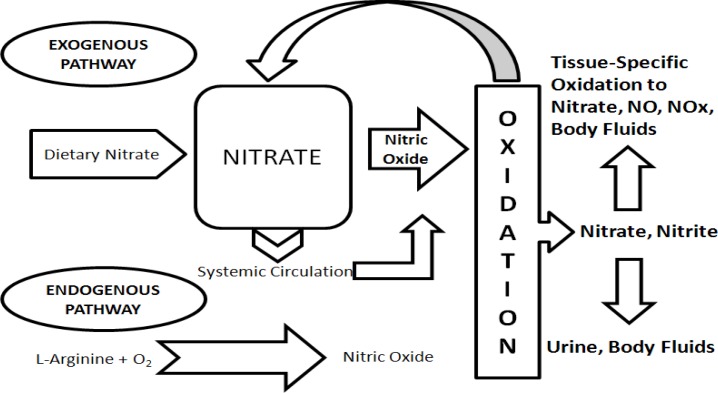
A schematic diagram of the physiological disposition of nitrate, nitrite, and nitric oxide from exogenous (dietary) and endogenous sources

In a study on nine (seven males, two females) healthy volunteers, nitrate ingestion resulted in a significant reduction of VO_2_ in sub-maximal work rates (Larsen, et al., 2010 ▶) without an increase in the blood lactate level, indicating that energy production had become more efficient. The mechanism is still not known but Larsen et al. speculated that NO or a related species may reduce the proton leakage over the mitochondrial membrane. Nitrite is a biologically active compound generated from nitrate reduction in tissues. A significant physiological benefit may be related with the absorption of nitrite from dietary sources (Hord, et al., 2009 ▶) (Figure 1). It is hypothesized that Nitric Oxide has a potential to promote an increase in blood flow to the active muscles and then leads to an increase in nutrient delivery to and waste removal from the muscles, thereby enhancing the exercise performance and recovery (Bloomer et al., 2011 ▶). 

Nitrate supplementation in healthy human subjects has been studied (Larsen, et al., 2011 ▶) and it has been postulated that dietary inorganic nitrate increases the capacity for ATP synthesis in mitochondria isolated from muscle biopsy tissue. Increase in ATP production capacity occurs in the absence of any increase in mitochondrial content. With nitrate supplementation, mitochondrial proton leak was reduced and oxidative phosphorylation efficiency was increased. This finding was a proof that nitrates decrease energy waste, and effectively increase the amount of ATP production per unit of oxygen consumed. Following a series of *in vitro *experiments, Larsen et al. showed that whole body oxygen expenditure during steady-state exercise decreases with nitrate supplementation while the mechanical work output to oxygen expenditure (Watt/VO_2_) concomitantly increases. Exercise economy can be affected by changes in mechanical efficiency, mitochondrial coupling efficiency or both. A short-term dietary nitrate supplementation enhances mitochondrial efficiency, decreases mitochondrial proton leaks, and enhances exercise performance (Nair, et al., 2011 ▶). Supplementation with 500 ml of beetroot juice for 6 consecutive days in eight healthy men was reported a significant reduction of O_2_ cost during cycling exercise at fixed submaximal cycling work rates (Bailey, et al., 2009 ▶). 

The author suggested that only a high dose of dietary nitrate (0.033 mmol NaNO_3_/kg three times daily) may be needed for performance improvements by possible improvement in the energy efficiency of active muscles. 

Current evidences supporting the notion that whole-body oxygen cost may be reduced because of improved mitochondrial efficiency. However, the underlying mechanisms are not clear and further studies are needed to rule out the possibility that nitrates may induce a shift toward non-oxidative pathways for cellular ATP synthesis. Such a shift may have negative consequences in tissues such as cardiac muscle, which relies heavily on oxidative metabolism to meet metabolic demands at rest and during exercise. However, extrapolation from skeletal muscle with mixed fiber types to myocardium with predominantly mitochondrial rich muscle fibers requires additional studies. Yet another study examined the effect of 500 ml of beetroot juice for 6 days in 6 maximal 500-m rowing-ergometer repetitions in fourteen well-trained junior male rowers without control group and best results found in the repetitions 4-6 in rowing time (1.7%, 95% CL, ± 10.0%) (Bond, et al., 2012 ▶).

Collectively, nitric oxide-stimulating dietary supplements are arguably the most widely advertised in the sports nutrition market. While some anecdotal reports suggest a potential benefit from using these products, some publications are failed to find established positive effect on exercise performance or physiological parameters. Reviewing all the published articles in this field indicate significant differences between the methodology, supplementation dose and data processing. 

According to a research in Tabriz of Iran, higher nitrate concentration was found in the tuberous vegetables especially in the leafy vegetables than fruit bearing vegetables (Tabatabaei, 2005 ▶). However, nitrate concentration in the high concentrate nitrate vegetables reported very different depending the cultivation and environmental factors. Red radish and watercress reported to be one of the medium to high nitrate vegetables but no study examined the effect of these vegetables on the oxygen consumption invitro or invivo. Nitrate concentration in the natural products classified in very low (<20 mg/100 g), low (20-50 mg/100 g), moderate (50-100 mg/100 g), high (100-250 mg/100 g), and very high (>260 mg/100 g) (Williams, 2012 ▶). 

Despite the lack of scientific evidences in the effectiveness of watercress and red radish on the exercise performance, the moderate concentration of nitrate in these vegetables afforded the opportunity to investigate the effect of watercress and red radish on the rest and in a graded exhaustive treadmill exercise by using extensive gas exchange data analysis. 

## Materials and Methods

**Participants**

Thirty-six healthy and non-active male university students volunteered to take part in a pre-test/post-test study. Participants randomly divided into three groups. They asked to refrain from any medium to vigorous exercise and their diets were controlled during the study by using a 24 h recall questionnaire. All subjects received written information about the aims of the study and signed an informed consent form. Ethical approval to conduct this study obtained from the University Human Ethics Committee. A sample of vegetables in each day was dried and finally analyzed to determine nitrate concentrations. 

**Supplementation**

Group 1 (n=11, age: 20.2±1.62 years, body mass: 69.0±13.4 kg) consumed 100 g fresh watercress, group 2 (n=11, age: 20.5±2.25 years, body mass: 70.6±15.3 kg) received 100 g fresh red radish and control group (n=14, age: 20.5±1.40 years, body mass: 70.3±10.8 kg) was prohibited from consumption of moderate dose of high-nitrate foods for seven days. Subjects were eaten the vegetables in the lab under supervision of the researcher at 11-12 a.m.

**Methodology**

One day before and after seven-day supplementation period, blood pressure, breath rate, heart rate, and three minutes respiratory gas analysis were recorded. All measurements were conducted after 30 min rest.

All subjects underwent standard treadmill exercise testing (Bruce protocol) (Bruce, et al., 1974 ▶) controlled by a treadmill control software developed by the author on a Lode treadmill (Valiant, Lode BV, The Netherlands) at 4 pm (three hours after meal). A heart rate transmitter belt (Polar Electro, Finland) was attached to the chest to transmit the heart rate signals to the treadmill’s receiver and then recorded in a database by the treadmill control software. 

**Data processing**

Inhaled O_2_, exhaled CO_2_, and ventilation rate were analyzed on a breath-by-breath basis using a respiratory gas analyzer using a calibrated Power Cube Ergo Gas Analyzer (Ganshorn Medizin Electronic GmbH, Nie derlauer, Germany). Gas exchange variables are recorded every ten seconds including: oxygen uptake (VO_2_), carbon dioxide production (VCO_2_), minute ventilation (VE), hear rate (HR), respiratory rate (RR), end-tidal oxygen tension (PETO_2_), end-tidal carbon dioxide tension (PETCO_2_), respiratory gas-exchange ratios (RER), oxygen uptake per heartbeat (O_2_-Pulse), ventilatory equivalents for O_2_ (EQO_2_), ventilatory equivalents for CO_2_ (EQCO_2_), tidal volume (VT), alveolar ventilation (VA), fractional end-tidal O_2_ concentration (FETO_2_), fractional end-tidal CO_2_ concentration (FETCO_2_), metabolic equivalent (METS), energy expenditure (EE), energy expenditure from carbohydrate (CHO EE), energy expenditure from fat (FAT EE).

Computer software was developed to calculate the total oxygen consumption, CO_2_ production, total ventilation volume, and O_2_-Pulse during the rest and exercise. Software computed the linear distance, vertical distance using the speed and treadmill incline every ten seconds. Distance is a function of time and speed of the treadmill. Work is calculated as the product of body mass (kg), gravity (9.81 m/s^2^), vertical speed (m/s) and slope, and time (s). Power is the product of bodyweight (kg), gravity (9.81 m/s^2^), and vertical speed (m/s) and slope.

The software automatically calculated the heart rate deflection point base on the method of D_max _(Siahkouhian and Meamarbashi, 2013 ▶) to estimate the anaerobic threshold. RER was also used to determine anaerobic threshold (AT). RER-AT point was determined when the RER stabilized above 10.0 and not returned under 10.0 (Dickstein, et al., 1990 ▶, Yeh, et al., 1983 ▶). Maximum values in the raw data were calculated throughout the tests. The software provided facilities to calculate maximum values during, integer values, normalized values for each subject in each session and create professional databases for data analysis by the SPSS software. 

To assess the effectiveness of watercress and red radish compare to the control group during aerobic or anaerobic phase of exercise, the maximum values before or after RER-AT point were compared. Additionally, to examine the effect of nitrate on the physiological parameters, pre-test and post-test duration was compared and only data from start until the equal time were processed. In the next step, maximum values in the equal time were normalized by the subject’s body mass. 

Transient spikes in the physiological parameters during the test may yield a false interpretation by the statistical methods. To overcome this miscalculation, by numerical integration the area under the parameter-time curve was computed by the software using a well-known trapezoidal rule method.

**Nitrate analysis**

Dried vegetables were powdered by grinding and 0.4 g powder was added to 40 ml aluminum sulfate 0.025 M. The solution was then filtered through a Whatman #42 filter paper. Ion-selective electrode (Metrohm, Switzerland) was used with nitrate electrode to measure the nitrate concentration.

**Statistical analyses**

Normal distribution was tested using the Kolmogorov-Smirnov and Shapiro-Wilk tests. Analysis of covariance (ANCOVA) with Bonferroni pairwise comparisons used to compare the pre-test and post-test in the experimental and control groups. The statistical analysis was performed using the Statistical Package for Social Sciences software (SPSS Version 16, SPSS Inc. Chicago, IL).

## Results

The Kolmogorov-Smirnov and Shapiro-Wilks tests revealed normality of the data. Raw watercress and red radish analysis determined 1095 and 1732 mg/kg nitrate respectively are considered high concentrate nitrate vegetables (1000-2500 mg/kg). This amount is approximately 5 to 7.8 times of Acceptable Daily Intake (ADI) for nitrate in human (3.7 mg/kg B.w./day) (EFSA, 2008 ▶). 

Blood pressure in the rest period was statistically analyzed. There was a significant reduction in the systolic blood pressure (p<0.001) and diastolic blood pressure in the red radish (p<0.01). In the watercress group, only a significant decrease found in the diastolic blood pressure (p<0.05) (Table 1).

To find out any physiological changes after seven-days consumption of watercress and red radish on the physiological parameters in the rest condition prior to the exercise, values obtained during the three minutes of gasometry were compared between groups. There were no any significant changes in the post-test as compared to pre-test between experimental groups in comparison with a control group using ANCOVA (Table 1).

**Table 1:** Physiological parameters obtained in Pre-Test and Post-Test during rest period (mean ± SD)

**NAME**	**Watercress**		**Red Radish**		**Control**	
	**Pre-Test**	**Post-Test**	**Pre-Test**	**Post-Test**	**Pre-Test**	**Post-Test**
**Resting Heart Rate**	85.0 ±13.1	81.5±13.5	91.0±12.7	83.9±12.3	76.0±14.3	81.5±14.27
**Resting Systolic BP**	118.5±9.0	116.5±14.6	125.0±7.6	115.5±5.4***	125.6±14.4	121.2±11.5
**Resting Diastolic BP**	73.7±8.7	67.9±8.6*	73.1±6.7	66.5±5.6**	70.7±7.5	65.2±7.5

Statistical difference between pre-test and post-test by (ANCOVA):

* p<0.05;

** p<0.01;

*** p<0.001.

Comparison between watercress and control groups during rest period showed a significant increase in the maximum values of RER (p<0.05), FETO_2_ (p<0.05), and energy consumption of carbohydrate (p<0.01). Comparison of red radish with control group indicated a significant increase in the VCO_2_ (p<0.01), RER (p<0.01), VT (p<0.05), VCO_2_/kg (p<0.05), and energy consumption of carbohydrate (p<0.01). Results in the Table 2 show a non-significant difference in the exercise time, work and power indicating no significant effect of nitrate on the exercise performance. To elucidate any physiological change after consumption of watercress and red radish during graded exercise (Bruce test), exercise duration of the pre-test was compared with the post-test and then all values in the equal time and normalized by the subject’s body mass were statistically analyzed (Table 3).

**Table 2:** Maximum gasometry values in the watercress, red radish and control groups in the Pre-Test and Post-Test during exercise (mean ± SD)

**Name**	**Watercress**		**Red Radish**		**Control**	
	**Pre-Test**	**Post-Test**	**Pre-Test**	**Post-Test**	**Pre-Test**	**Post-Test**
**Time [Sec]**	808.1 ± 76.92	840.0 ± 102.7	829.0 ± 76.8	846.3 ± 84.0	761.4 ± 81.51	770.7 ± 72.4
**Speed [Km/h]**	7.83 ± 0.610	8.02 ± 0.544	7.95 ± 0.480	8.10 ± 0.592	7.62 ± 0.609	7.53±0.646
**Incline [%]**	17.8 ± 1.08	18.1 ± 1.08	18.0 ± 0.89	18.3 ± 1.21	17.4 ± 0.94	17.2 ± 0.99
**Work [J]**	106.8 ± 17.0	114.6 ± 21.0	114.6 ± 19.1	120.0 ± 21.8	100.9 ± 30.0	101.3 ± 26.4
**Power [W]**	0.13 ± 0.019	0.137 ± 0.018	0.139 ± 0.023	0.142 ± 0.024	0.130 ± 0.028	0.130 ± 0.026
**Distance [m]**	1122±174.8	1195 ± 235.3	1165 ± 159.3	1210 ± 186.2	1024 ± 170.5	1037 ± 150.2
**Vertical Distance [m]**	161.5 ± 32.2	174.9 ± 44.0	169.3 ± 28.3	177.6 ± 33.9	144.3 ± 29.5	146.4 ± 26.4
**Total O**_2_ ** (L)**	24.0 ± 3.74	24.9 ± 4.43	25.0 ± 4.88	26.0 ± 5.28	22.3 ± 5.93	21.3 ± 5.47
**Total CO**_2_ ** (L)**	26.7 ± 4.15	28.6 ± 5.46	28.2 ± 5.12	30.2 ± 5.89	25.3 ± 7.04	23.2 ± 6.03
**Total Ventilation (L)**	743.8 ± 157.2	795.7 ± 204.0	767.1 ± 161.8	791.0 ± 183.4	659.7 ± 184.0	608.2 ± 151.3
**VO**_2_ ** [L/min]**	3.13 ± 0.404	3.12 ± 0.321	3.15 ± 0.433	3.22 ± 0.470	3.04 ± 0.539	3.05 ± 0.527
**VCO**_2_ ** [L/min]**	4.16 ± 0.516	4.22 ± 0.470	4.27 ± 0.621	4.46 ± 0.667*	4.08 ± 0.812	3.92 ± 0.669
**VE [L/min]**	128.5 ± 22.7	130.5 ± 20.1	127.5 ± 18.74	129.5 ± 24.5	113.1 ± 26.7	108.3 ± 20.2
**HR [1/min]**	201.4 ± 8.20	202.6 ± 9.58	204.3 ± 8.76	201.3 ± 9.15	195.0 ± 9.41	193.3 ± 9.30
**RR [1/min]**	54.4 ± 7.50	53.8 ± 7.87	55.6 ± 9.47	53.4 ± 9.41	49.0 ± 6.17	47.8 ± 6.17
**PETO**_2_ ** [mmHg]**	98.4 ± 3.26	99.6 ± 3.71	98.6 ± 3.10	98.3 ± 4.04	96.8 ± 2.83	95.9 ± 2.33
**PETCO**_2_ ** [mmHg]**	42.9 ± 3.15	43.4 ± 3.00	45.1 ± 5.29	46.1 ± 4.25	45.4 ± 4.63	44.6 ± 2.84
**RER**	1.33 ± 0.043	1.35 ± 0.036*	1.35 ± 0.056	1.39 ± 0.084*	1.34 ± 0.106	1.29 ± 0.078
**VO**_2_ **/KG [ml/kg]**	46.0 ± 5.56	46.3 ± 5.75	45.6 ± 6.12	46.6 ± 6.12	43.2 ± 4.20	43.5 ± 3.37
**VCO**_2_ **/KG [ml/kg]**	61.4 ± 8.31	62.5 ± 8.26	61.7 ± 8.25	64.3 ± 7.26*	57.8 ± 6.70	56.0 ± 6.18
**VE/KG [ml/kg/min]**	1902 ± 400.5	1952 ± 453.4	1859 ± 368.6	1877 ± 339.9	1598 ± 240.4	1546 ± 187.2
**O**_2_ **-PULSE [ml/beat]**	0.016 ± 0.002	0.016 ± 0.002	0.016 ± 0.002	0.017 ± 0.003	0.016 ± 0.003	0.016 ± 0.003
**EQO**_2_	40.1 ± 4.75	41.3 ± 5.85	40.2 ± 4.88	39.9 ± 5.87	36.7 ± 3.79	35.3 ± 2.92
**EQCO**_2_	31.8 ± 3.76	32.2 ± 4.10	33.0 ± 3.61	31.2 ± 2.53	31.2 ± 3.62	30.4 ± 3.25
**VT [L]**	2.44 ± 0.454	2.53 ± 0.420	2.37 ± 0.474	2.50 ± 0.567*	2.41 ± 0.427	2.34 ± 0.381
**VA [L]**	128.5 ± 22.8	130.5 ± 20.1	127.4 ± 18.7	129.5 ± 24.5	113.0 ± 26.7	108.4 ± 20.3
**FETO**_2_ ** [%]**	16.2 ± 0.54	16.4 ± 0.61*	16.2 ± 0.51	16.2 ± 0.67	16.0 ± 0.47	15.7 ± 0.38
**FETCO**_2_ ** [%]**	7.08 ± 0.518	7.18 ± 0.493	7.45 ± 0.871	7.62 ± 0.702	7.51 ± 0.765	7.32 ± 0.465
**METS [kcal/kg/h]**	13.1 ± 1.58	13.2 ± 1.64	13.0 ± 1.75	13.3 ± 1.74	12.3 ± 1.20	12.4 ± 0.97
**EE [Kcal/h]**	1006 ± 127.6	1009 ± 104.1	1020 ± 141.4	1048 ± 150.5	981.0 ± 176.4	973.4 ± 165.1
**CHO EE [Kcal/h]**	2076 ± 265.9	2151 ± 275.0*	2177 ± 353.4	2326 ± 417.5*	2056 ± 516.7	1877 ± 365.8
**FAT EE [Kcal/h]**	117.4 ± 61.6	87.0 ± 72.6	125.5 ± 117.1	88.5 ± 76.3	106.2 ± 71.0	132.3 ± 71.4

Significant difference compare with control group obtained by ANCOVA:

* p<0.05

Normalized values by the subject’s body mass in the same exercise duration and work load in the watercress group had a significant increase in the total CO_2_ production (p<0.05), RER (p<0.05), FETO_2_ (p<0.05), and energy consumption of carbohydrate (p<0.05) compared to the control group. Red radish comparison with control group revealed a significant increase in pre-test in the total CO_2_ production (p<0.05), VCO_2_ (p<0.05), RER (p<0.01), VT (p<0.05), VCO_2_/kg (p<0.05) (Table 3).

It is important to know when integer values of all the parameters during the test were compared and analyzed, no significant changes were found in the VO_2_ and ventilation rates. Body mass was not different between pre-tests and post-test in the groups.

Data before and after the anaerobic threshold in the RER-AT point was compared with the same phase in the post-test showed no significant difference between the parameters in the watercress or red radish as compared with control. 

**Table 3:** Maximum gasometry values in the watercress, red radish and control groups in the Pre-Test and Post-Test during exercise in equal time and normalized by the subject’s body mass (Mean ± SD)

**NAME**	**Watercress**		**Red Radish**		**Control**	
	**Pre-Test**	**Post-Test**	**Pre-Test**	**Post-Test**	**Pre-Test**	**Post-Test**
**VO**_2_ ** [L/min]**	0.036 ± 0.005	0.035 ± 0.004	0.036 ± 0.005	0.036 ± 0.005	0.034 ± 0.006	0.034 ± 0.006
**VCO**_2_ ** [L/min]**	0.047 ± 0.006	0.047 ± 0.006	0.048 ± 0.008	0.049 ± 0.008*	0.046 ± 0.009	0.043 ± 0.008
**Work [J/Kg BW]**	1.21 ± 0.194	1.19 ± 0.189	1.27 ± 0.250	1.26 ± 0.240	1.11 ± 0.324	1.09 ± 0.320
**Power [W/Kg BW]**	0.002 ± 0.000	0.001 ± 0.000	0.002 ± 0.000	0.002 ± 0.000	0.001 ± 0.000	0.001 ± 0.000
**Distance [m/Kg BW]**	12.7 ± 1.99	12.6 ± 1.95	12.9 ± 1.82	12.8 ± 1.78	11.4 ± 1.91	11.2 ± 1.84
**Vertical Distance [m/Kg BW]**	1.83 ± 0.366	1.81 ± 0.360	1.87 ± 0.322	1.86 ± 0.316	1.60 ± 0.332	1.57 ± 0.319
**RER**	0.015 ± 0.000	0.015 ± 0.000*	0.015 ± 0.001	0.015 ± 0.001*	0.015±0.001	0.014 ± 0.001
**VO**_2_ **/KG [ml/kg]**	0.524 ± 0.063	0.516 ± 0.062	0.513 ± 0.069	0.520 ± 0.072	0.487 ± 0.048	0.484 ± 0.040
**VCO**_2_ **/KG [ml/kg]**	0.698 ± 0.094	0.691 ± 0.086*	0.690 ± 0.097	0.705 ± 0.092*	0.649 ± 0.078	0.616 ± 0.067
**Total O**_2_ ** (L/Kg BW)**	24.0 ± 3.74	23.3 ± 3.72	24.5 ± 5.63	24.5 ± 5.15	21.8 ± 5.59	20.5 ± 5.78
**Total CO**_2_ ** (L/Kg BW)**	26.7 ± 4.15	26.4 ± 4.34*	27.6 ± 6.10	28.1 ± 5.71*	24.6 ± 6.66	22.1 ± 6.24
**Total Ventilation (L/Kg BW)**	743.8± 57.2	724.5 ± 157.5	745.7 ± 190.5	729.4 ± 158.7	638.9 ± 164.7	578.1 ± 159.3
**METS [kcal/kg/h/Kg BW]**	0.150 ± 0.018	0.148 ± 0.018	0.146 ± 0.020	0.149 ± 0.020	0.139 ± 0.014	0.138 ± 0.011
**FETO**_2_ ** [%]**	0.185 ± 0.006	0.183 ± 0.007*	0.184 ± 0.005	0.180 ± 0.008	0.181 ± 0.005	0.175 ± 0.003
**VT [L]**	0.028 ± 0.005	0.028 ± 0.005	0.026 ± 0.006	0.028 ± 0.007*	0.027 ± 0.005	0.026 ± 0.004
**EE [Kcal/h/Kg BW]**	11.4 ± 1.45	11.2 ± 1.26	11.4 ± 1.79	11.6 ± 1.73	11.0 ± 1.94	10.8 ± 1.95
**CHO EE [Kcal/h/Kg BW]**	23.6 ± 3.02	23.5 ± 3.30*	24.2 ± 4.58	24.9 ± 4.65	22.9 ± 5.72	20.4 ± 4.16
**FAT EE [Kcal/h/Kg BW]**	1.33 ± 0.700	0.97 ± 0.816	1.42 ± 1.332	0.99 ± 0.858	1.20 ± 0.807	1.48 ± 0.803

Significant difference compare with control group obtained by ANCOVA:

* p<0.05.

## Discussion

After extensive data analysis on the gas exchange values, heart rate and exercise duration, work, power, and other parameters in the current study despite some significant finings in the gas exchange values, major finding was increase in the total CO_2_ production at the equal time of exercise and normalized by the subject’s body mass. Our findings showed a negative impact of watercress and red radish on exercise performance due to increase in the CO_2_ production without an increase in ventilation. Besides, current findings do not support recent findings of Larsen et al. (2010 ▶, 2011), Vanhatalo et al. (2010) ▶, Lansley et al. (2010) ▶, Lee et al. (2010), Bailey (2009) ▶, Vanhatalo et al. (2010) ▶ that claimed the reduction in the oxygen consumption during exercise despite differences in supplementation, duration and exercise methods. However, the beneficial effects of nitrate has been argued (Bloomer, 2010 ▶) and current findings are demanding more attention in processing the raw data. It must be noted that all the aforementioned reports compared the rate of oxygen consumption (VO_2_), but it is not conceivable parameter to evaluate oxygen cost of exercise when the total oxygen volume has not been calculated in the same exercise workload. Extensive data processing by the custom design software is an advantage of this research to unravel any mechanistic effect of supplementation on the resting metabolism or moment by moment changes in the exercise performance.

Beetroot juice (500 ml/day) was mostly used in the previous investigations (Bailey, et al., 2009 ▶, Lansley, et al., 2011 ▶, Vanhatalo, et al., 2010 ▶) providing 340 mg nitrate which is about three times of the dose used in this study. Significant reductions in the resting blood pressure may indicate the physiological effect of nitrate in our research. However, in this phase (rest) no significant changes was found in the pulmonary gas exchange values.

In the current research, seven days of supplementation with moderate dose of high-nitrate vegetables (watercress and red radish) in non-active young male subjects caused an increase in the total CO_2_ production without significant changes in the pulmonary ventilation. 

This phenomenon induces metabolic acidosis that menaces the exercise performances. Therefore, the impact of supplementation with high-nitrate foods should be cautiously studied considering appropriate dosing, washout period in the cross over designs, supplementation duration, method of exercise level, and data processing strategy.
